# Effects of different aerobic exercise training on glycemia in patients with type 2 diabetes: A protocol for systematic review and meta analysis

**DOI:** 10.1097/MD.0000000000025615

**Published:** 2021-05-07

**Authors:** Ying Li, Runmin Li, Xianhuang Li, Liping Liu, Jianting Zhu, Dongying Li

**Affiliations:** a Intensive Care Unit, The Second Affiliated Hospital of Nanchang University, Nanchang City, Jiangxi Province, Nanchang; b College of Traditional Chinese Medicine, Shandong University of Traditional Chinese Medicine, Jinan; c School of Nursing, Nanchang University, Jiangxi, People's Republic of China.

**Keywords:** aerobic exercise training, mesh meta-analysis, type 2 diabetes

## Abstract

**Background:**

Type 2 diabetes is an emergent worldwide health crisis, and rates are growing globally. Aerobic exercise is an essential measure for patients with diabetes, which has the advantages of flexible time and low cost. Aerobic exercise is a popular method to reduce blood glucose. Due to the lack of randomized trials to compare the effects of various aerobic exercises, it is difficult to judge the relative efficacy. Therefore, we intend to conduct a network meta-analysis to evaluate these aerobic exercises.

**Methods:**

According to the retrieval strategies, randomized controlled trials on different aerobic exercise training will be obtained from China National Knowledge Infrastructure, WanFang, SinoMed, PubMed, Web of Science, EMBASE, and Cochrane Library, regardless of publication date or language. Studies were screened based on inclusion and exclusion criteria, and the Cochrane risk bias assessment tool will be used to evaluate the quality of the literature. The network meta-analysis will be performed in Markov Chain Monte Carlo method and carried out with Stata14 and OpenBUGS software. Ultimately, the evidentiary grade for the results will be evaluated.

**Results:**

Eighteen literatures with a total of 1134 patients were included for the meta-analysis. In glycemia assessment, Tennis (standard mean difference = 3.59, credible interval 1.52, 5.65), had significantly better effects than the named control group. Tennis (standard mean difference = 3.50, credible interval 1.05, 5.59), had significantly better effects than the named Taiji group.

**Conclusion:**

All together, these results suggest that tennis may be the best way to improve blood glucose in patients with type 2 diabetes. This study may provide an excellent resource for future control glycemia and may also serve as a springboard for creative undertakings as yet unknown.

## 1 Introduction

Type 2 diabetes is an emergent worldwide health crisis, and rates are growing globally.^[1–3]^ More than 500 million people in 45 countries worldwide are effected by diabetes, in 2018.^[4]^ By 2045, an estimated 629 million people will have this lifelong chronic diseases.^[5]^ It was estimated that 114 million adults were living with diabetes in 2017, which is expected to rise to 120 million by 2045 in china.^[6]^ Type 2 diabetes mellitus (T2DM) increases the risk of cardiovascular disease, neuropathy, nephropathy, retinopathy, and microvascular complications. Moreover, it has been reported that the risk of heart disease among diabetic patients is 2- to 4-fold higher compared with normal subjects.^[7]^ In the word, disability caused by type 2 diabetes is increasing.^[8]^ In 2017, diabetes has become the fourth leading cause of disability globally.^[9]^ In addition, the economic burden of diabetes is also huge. The global cost of diabetes is $1.31 trillion, accounting for 1.8% of global GDP.^[10]^ Therefore, fighting diabetes mellitus has become an urgent challenge.

T2DM is a metabolic disorder characterized by hyperglycemia and a chronic and progressive metabolic state characterized by hyperglycemia.^[11]^ Insulin damage, insulin resistance in peripheral tissues, or a combination of the 2 are the most common pathophysiological causes of T2DM.^[11]^ In addition, the plasma levels of adipokines and resistin in patients with type 2 diabetes may be increased, which may also increase impaired glucose tolerance and insulin resistance.^[12]^ The American Diabetes Association recommends 2 types of exercise mode for individuals with diabetes, which includes strengthening exercises and aerobic exercises.^[13]^ Aerobic exercise is the basis of the treatment of type 2 diabetes, and it is an indispensable part of the comprehensive treatment of type 2 diabetes.

Aerobic exercise refers to repeated and continuous exercise of large muscle groups, including walking, cycling, jogging, and swimming.^[14,15]^ The study found that regular aerobic exercise can not only reduce glycosylated hemoglobin, improve cardiopulmonary function, enhance lipid oxidation, increase aerobic fitness ability, promote liver glucose metabolism, but also reduce the mortality of diabetes.^[16–18]^ In addition, aerobic exercise also has economic benefits, simple, unrestricted, and other advantages.

Early aerobic exercise is very necessary for patients with type 2 diabetes. However, there are many types of aerobic exercise styles in T2DM, and little attention has been paid to the ranking of aerobic exercise effectiveness. Network meta-analysis (NMA) is a new method that employs Bayesian statistical theory.^[19]^ Although aerobic exercise, such as Taijiquan, jogging, and swimming, is generally believed to be beneficial to patients with type 2 diabetes, the above interventions are hardly measurable. To address this question, Bayesian NMA enables a comprehensive analysis through integrating all direct and indirect evidence to compare various interventions.^[20]^ In the mesh meta-analysis, the accuracy of effect estimation is improved than that of traditional meta-analysis, which allows the estimation of direct and indirect therapeutic effects.^[21]^ Therefore, the main purpose of this study is to find out the best aerobic exercise mode for patients with type 2 diabetes by summarizing and analyzing the existing evidence.

## 2 Methods

The literature search of the NMA is based on the expanded statement of preferred reporting items for systematic review and meta-analysis systematic evaluation report, including the NMA of health interventions.^[22]^ The retrieval strategy was formulated by 2 authors according to the research purpose. Without the limitation of year and language, a detailed search method of PubMed, Cochrane, and Web of Science was made by using medical subject words (mesh), text words, and Boolean logic operators Science, EMBASE, HowNet, Wanfang, etc. Taking PubMed as an example, the retrieval strategy is shown in Table 1. The retrieval time is from the database establishment to September 2020. The keywords are as follows: walking, brisk walking, jogging, swimming, cycling, Taijiquan, Baduanjin, etc. These terms use the operator “and” to combine with type 2 diabetes, noninsulin dependent diabetes, and T2DM.

**Table 1:** Retrieval strategy of PubMed.

#1	“Diabetes Mellitus, Type 2” [MeSH] OR “Diabetes Mellitus, Noninsulin-Dependent” [Title/Abstract]
	OR “Diabetes Mellitus, Non Insulin Dependent” [Title/Abstract]
	OR “Non-Insulin-Dependent Diabetes Mellitus” [Title/Abstract] OR “Diabetes Mellitus, Type II” [Title/Abstract]
	OR “Diabetes Mellitus, Noninsulin Dependent” [Title/Abstract] OR “Diabetes Mellitus, Maturity-Onset” [Title/Abstract]
	OR “Diabetes Mellitus, Maturity Onset” [Title/Abstract] OR “Diabetes Mellitus, Slow-Onset” [Title/Abstract]
	OR “Slow-Onset Diabetes Mellitus” [Title/Abstract] OR “Type 2 Diabetes Mellitus” [Title/Abstract]
	OR “Noninsulin-Dependent Diabetes Mellitus” [Title/Abstract] OR “Type 2 Diabetes” [Title/Abstract]
	OR “Noninsulin Dependent Diabetes Mellitus” [Title/Abstract] OR “Maturity-Onset Diabetes” [Title/Abstract]
	OR “Diabetes, Type 2” [Title/Abstract] OR “Diabetes Mellitus, Adult-Onset” [Title/Abstract]
	OR “Adult-Onset Diabetes Mellitus” [Title/Abstract] OR “Diabetes Mellitus, Non Insulin Dependent” [Title/Abstract]
#2	Training, Resistance” [Title/Abstract]OR “Walking” [Title/Abstract]OR “Ambulation” [Title/Abstract]
	OR “Jogging” [Title/Abstract] OR “Bicycling”[Title/Abstract] OR “Taiji boxing” [Title/Abstract]
	OR “Aerobic exercise” OR “Physical Activity” [Title/Abstract] OR “Physical Activity” [Title/Abstract]
	OR “Exercise, Isometric” OR “Exercise, Isometric” [Title/Abstract] OR “Exercise, Aerobic” [Title/Abstract]
	OR “Aerobic Exercise” OR “Aerobic Exercises” [Title/Abstract] OR “Exercises, Aerobic” [Title/Abstract]
#3	Randomized controlled trial [Title/Abstract] OR Controlled clinical trial [Title/Abstract].
#4	#1 AND #2 AND #3

### 2.1 Objectives and registration

This systematic review will aim to evaluate the effectiveness of aerobic exercise in reducing fasting blood glucose. Our protocol has been registered on the International Platform of Registered Systematic Review and Meta-Analysis Protocols (INPLASY). The registration number was INPLASY202130055 (DOI:10.37766/inplasy2021.3.0055).

### 2.2 Ethics and communication plan

Our article is a secondary study, which does not involve the recruitment of patients, data collection, and ethical considerations. We will publish the results of NMA in the form of journal papers or conference papers.

### 2.3 Qualification and exclusion criteria qualification criteria

Participants: adult patients with T2DM. The diagnostic criteria of T2DM were consistent with those of who, American Diabetes Association or Chinese Diabetes Association.^[23–25]^ Interventions: participate in at least 1 aerobic exercise, such as walking, jogging, swimming, cycling, Taijiquan, Baduanjin. Comparison: routine life or other activities. Results: the main outcome was glycemia in patients with type 2 diabetes. Study design: randomized controlled trials (RCTs) were conducted in adults with T2DM. The exclusion criteria were as follows:

(1) animal experiments;
(2) non-RCTs, reviews or meta-analysis;
(3) incomplete information in published articles, which only presented in abstract form;
(4) other forms of exercise other than aerobic exercise.

### 2.4 Study selection and data extraction

**Figure 1:**
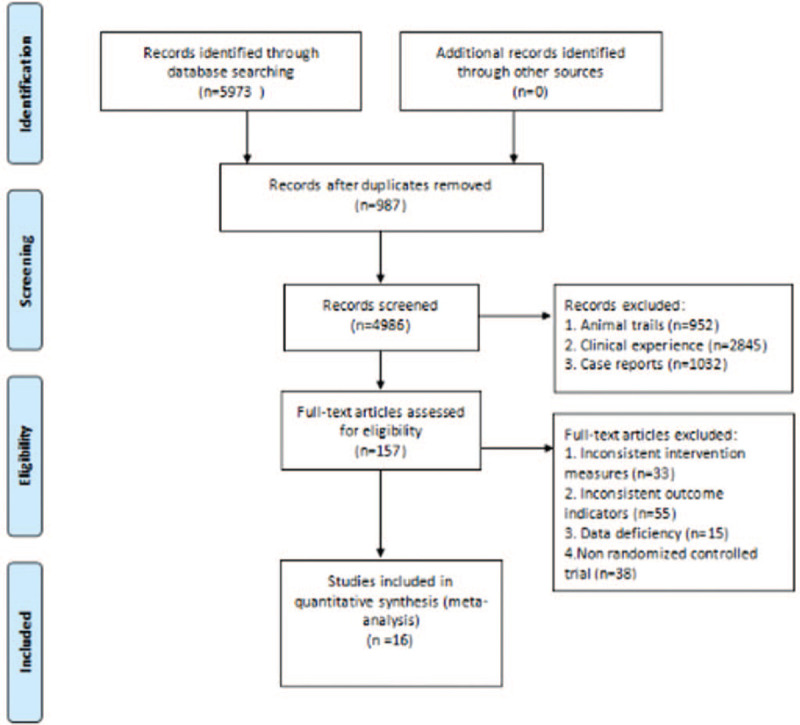
PRISMA flow chart. PRISMA = preferred reporting items for systematic review and meta-analysis.

As shown in Figure 1, the study selection will be divided into 2 steps and completed by 2 researchers (Xianhuang Li and Liping Liu). According to the criteria of Cochrane,^[23]^ an extraction table was established to extract information related to the study, including author information, study design, intervention measures, participant characteristics, outcome measurement, intervention duration, and other required information. If the data is not clear or there is objection to the data information, contact the author to verify and supplement the data. If there is any problem in the extraction process, the problem can be solved through consultation or consulting experts.

### 2.5 Deviation risk assessment

Two reviewers independently assessed and classified the included articles according to Cochrane Handbook,^[26]^ and classified them according to the established criteria (Xianhuang Li, Liping Liu). Finally, the study was divided into high risk, low risk, and uncertainty according to the allocation concealment, whether the subjects and researchers were blind, and whether they were random. In case of any disagreement or disagreement between the 2 reviewers in the process of evaluation, the 2 reviewers shall negotiate or consult experts (Ying Li, Xianhuang Li).

### 2.6 Statistical analysis

Bayesian NMA can summarize a variety of treatment measures, thus allowing maximum flexibility to use complex models and produce relatively scientific explanations in terms of causality.^[27]^ Bayesian NMA integrates direct evidence and indirect evidence into an interconnected network through prior distribution, so as to compare different aerobic exercise modes.

The network diagram serves as a simple summary description to reveal all available evidence for each treatment evidence.^[20]^ The network diagram is generated by Stata 14.0 (Stata Corp, College Station, TX). As the presence of effect sizes refer to continuous outcome, standard mean differences (SMDs) were calculated for each comparison using group (relevant) means and standard deviations from individual studies.^[28]^ We compared the clinical and methodological characteristics of all included studies in order to ensure sufficient similarity between different intervention comparisons, so as to provide effective indirect inference.^[29]^

In the Bayesian framework, the parameters are estimated by the restricted maximum likelihood method.^[30]^ We use Bayesian random effect model to integrate direct and indirect estimation by forming a connection network and comparing different aerobic exercise modes. We also use multivariate meta-analysis method to compare. In order to simulate the accurate estimation of statistical patterns, 3 parallel Markov chains were initially established with randomly selected states.^[31]^ Each chain generates 50,000 iterations. Due to the aging period, the first 20,000 iterations are discarded to ensure the minimum deviation of the initial value when the chain reaches its target distribution. The surface under the cumulative ranking curve (SUCRA) is represented as a simple statistical cumulative ranking probability graph, which is used to rank each intervention. The higher the SUCRA value is, the greater the possibility of a specific treatment being at the top or high efficiency, while 0 is the treatment that is definitely the worst.^[32]^ We use the “node splitting” technology to explore whether there will be potential source inconsistency in our network.^[33]^ By comparing the direct evidence and indirect evidence from the whole network (*P* > .05 indicates consistency generation),^[34]^ subgroup analysis is conducted if there is inconsistency. The above analysis was performed using “STATA14” (64 bit version) and OPENBUGES.

## 3 Results

A total of 5973 records were obtained. The title and abstract screening revealed 205 potentially qualified articles, of which 987 were repetitive and 4986 covered unique RCTs. Based on the full-text examination, 157 records were preliminarily consistent with the study: 33 including inconsistent intervention measures, 55 research reports with inconsistent outcome indicators, 34 were not RCTs, 15 studies were missing data, and 20 studies did not determine the control group. Finally, only 16 articles were considered qualified and contributed to our final NMA.^[35–50]^ The studies, which consisted of 1134 participants, provided sufficient data from the database establishment year to 2020. The basic characteristics of the included literature are shown in Table 2 .

**Table 2:**
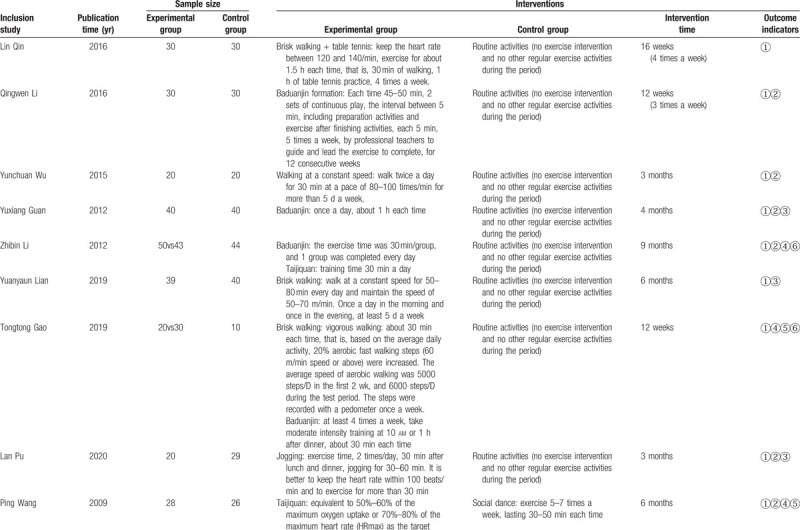
Basic information included in the study.

**Table 2 (Continued):**
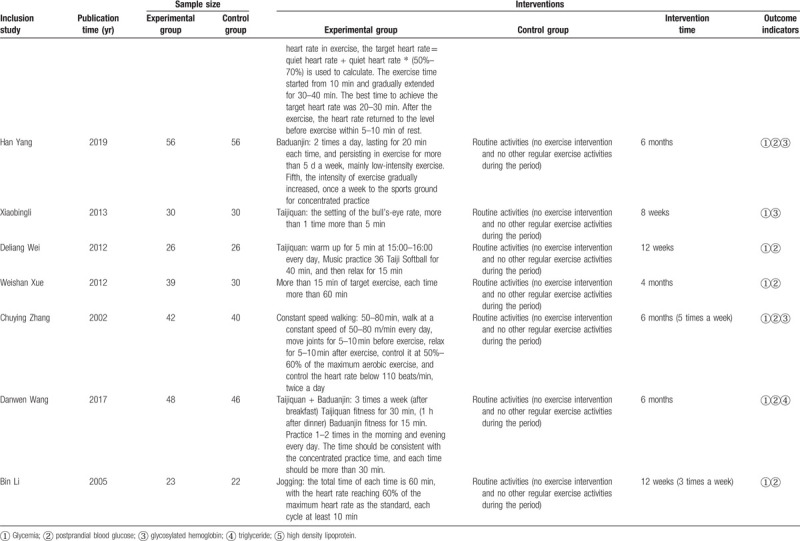
Basic information included in the study.

We conducted a global model test and a local inconsistency test for the articles. The degree of inconsistency in all studies was very small (*P* > .005). Depending on the distribution of the funnel in this publication, the distribution of these points is symmetric.

**Figure 2:**
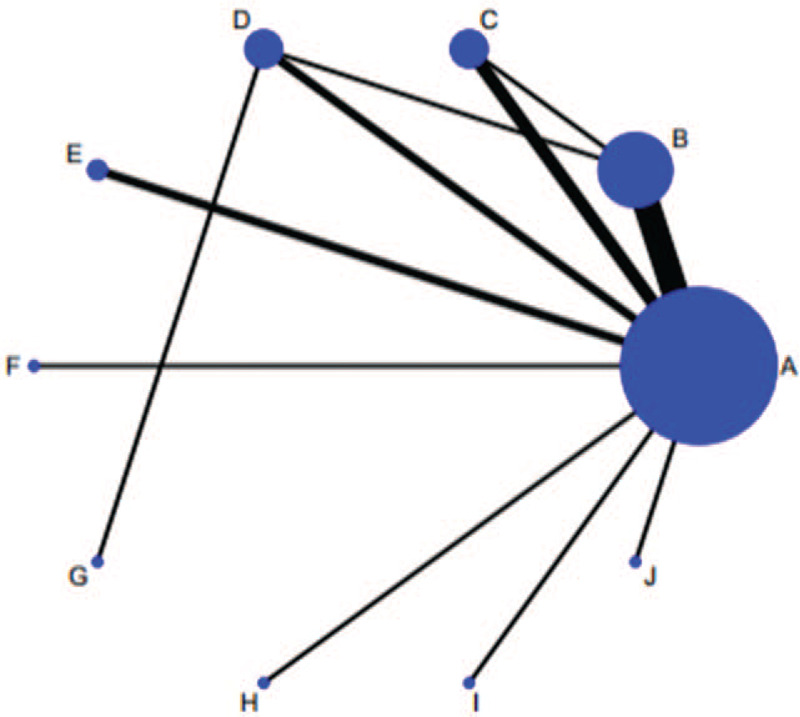
Network of evidence of all the trials.

As shown in Figure 2, conduct network diagram display and study the situation of each arm. Each process has its own unique node, and its size depends on the number of contributions they make to the entire network. We describe 16 comparisons in the type 2 diabetes group in the NMA. Baduanjin Exercise (n = 195) was the most frequent intervention, followed by constant speed walking (n = 99) and Taijiquan group (n = 99). Jogging group (n = 43), social dance group (n = 26), Taijiquan and Baduanjin group (n = 48), Taiji softball group (n = 26), tennis group (n = 30), vigorous walking and table tennis group (n = 30). Relative effect sizes of efficacy at postintervention according to NMA.

**Figure 3:**
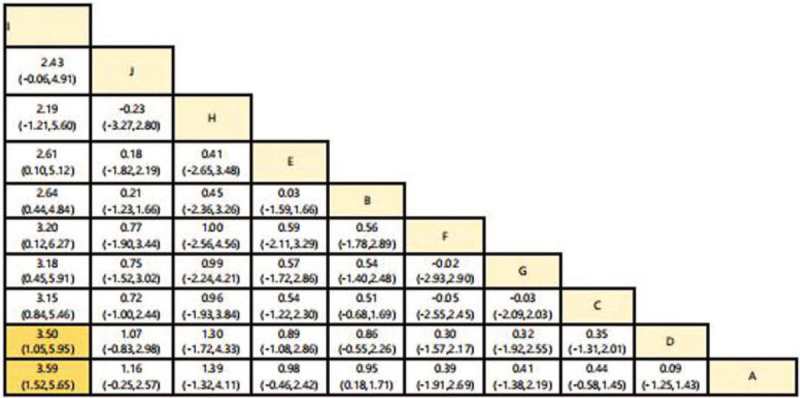
Relative effect sizes of efficacy at postintervention according to network meta-analysis.

**Figure 4:**
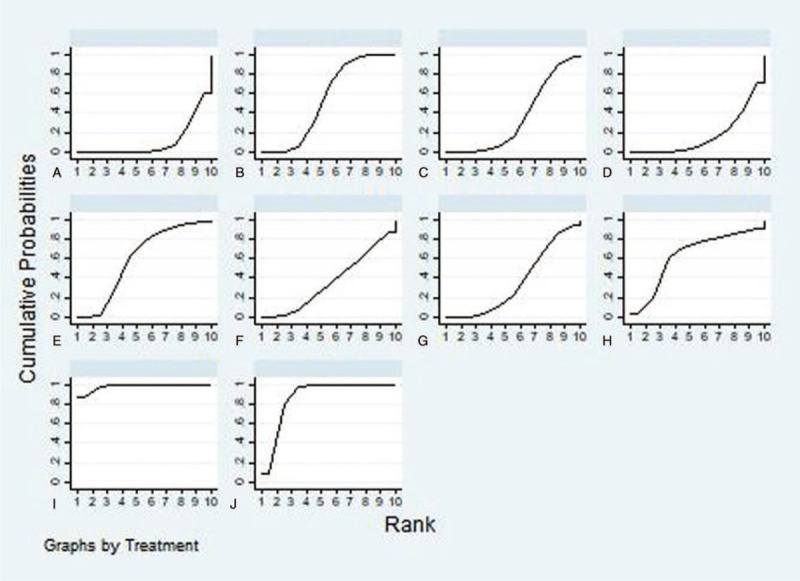
SUCRA plot. SUCRA = surface under the cumulative ranking curve.

Considering the efficacy of each intervention from baseline to the end of it, Tennis group was statistically significant superior to Routine activities group and Tai ji quan group (SMD = 3.59, credible interval 1.52, 5.65, (SMD = 3.50, credible interval 1.05, 5.95).(shown in Fig. 3). A SUCRA line was drawn to rank the hierarchy of each aerobic exercise (shown in Fig. 4), which indicated that Tennis got the highest probability (SUCRA = 98.4%) in reducing glycemia compared with the other 9 active interventions, although walking + table tennis (SUCRA = 87.3%) also got a remarkable ranking among the 10 treatments. Following by Taiji Soft ball (SUCRA = 63.5%), jogging SUCRA = (60.5%), Baduanjin (SUCRA = 54.6%), Taijiquan + Ba duan jin (SUCRA = 36.4%), social dance (SUCRA = 36.3%), constant walking (SUCRA = 35.6%), Taijiquan (SUCRA = 17.0%), and routine activities (SUCRA = 10.5%) got an inferior ranking.

## 4 Discussion

Despite some progress in the diagnosis and treatment of diabetes, patients with type 2 diabetes remain a major public health burden worldwide. Exercise is an essential measure for patients with diabetes, which has the advantages of flexible time and low cost. In this study, we conducted a meta-analysis of RCTs published so far. The results obtained in the league table show that only tennis and Taijiquan are 3.50 (1.05, 5.95), tennis and regular activities are 3.59 (1.52, 5.65), only 2 groups of data have difference. Taijiquan belongs to low-intensity exercise, and its peak exercise intensity only reaches about 45% of the maximum heart rate, so the metabolism of glucose and lipid in physiological function is weak.^[51]^ According to Colberg et al,^[52]^ type 2 diabetic patients use the most blood glucose during moderate or above intensity exercise, and the energy supply of myoglycogen oxidation is relatively less, thus reducing the blood glucose of patients with type 2 diabetes. Tennis belongs to moderate intensity exercise. Its physiological energy supply is mainly supplied by glycogen and supplemented by fat. It is superior to Taijiquan and routine activities in reducing glycemia.

In our study, we reviewed the effects of different aerobic exercise interventions on blood glucose in patients with type 2 diabetes. SUCRA analysis showed that tennis (98.4%) was the most effective intervention to reduce glycemia in patients with type 2 diabetes, followed by vigorous walking + table tennis (87.3%), Taiji soft ball (63.5%), jogging (60.5%), Baduanjin (54.6%), Taijiquan + Baduanjin (36.4%), social dance (36.3%), constant walking (35.6%), Taijiquan (17.0%), and routine activities (10.5%). Our research results are different from those of Ye Xinxin.^[53]^ In our research results, tennis is the best exercise measure to reduce glycemia in patients with type 2 diabetes, and tennis exercise mainly relies on muscle contraction and relaxation. Muscle contraction and relaxation can improve the tissue demand for sugar and improve the level of glucose metabolism. Long time exercise can not only promote the decomposition of glycosylated hemoglobin, but also improve the oxygen carrying level of blood, providing a good basis for the utilization of sugar.^[54]^ Brisk walking + table tennis cloud sports is only tennis, walking belongs to the lower limb movement, table tennis belongs to the upper limb movement, the combination of the 2 can achieve the whole body balanced movement.^[37]^ Long time but monotonous exercise can shorten the exercise time. Walking and table tennis are the combination of 2 aerobic exercises, which can avoid such defects and increase the exercise time.

To sum up, our network meta analysis shows that tennis is the best exercise intervention for patients with type 2 diabetes, while routine activities (without regular activities) are the least effective intervention. Our study may provide strong evidence to show which exercise intervention is the best intervention to reduce patients with type 2 diabetes in this unique population, and provide enlightenment for future research. Among the numerous aerobic exercises, tennis has achieved good results in reducing blood glucose in patients with type 2 diabetes.

## 5 Limitations

In view of the following limitations, we should carefully explain this study. First, this study contains a limited number of experiments. First, there are only a few RCT studies on tennis, brisk walking, brisk walking, and table tennis in the literature. Second, for diabetes, other outcome indicators are less involved. Third, the quality of several studies, such as lack of blindness in subject participation, personnel or external evaluators, or not using randomized grouping, may threaten the effectiveness of our study. For various reasons, so many aerobic exercise measures are not the result of RCTs, so the evidence based on aerobic exercise is limited, which makes it difficult to draw conclusions from our NMA.

## Author contributions

**Conceptualization:** Ying Li, Runmin Li, Dongying Li.

**Data curation:** Ying Li, Xianhuang Li, Liping Liu

**Formal analysis:** Ying Li and Runmin Li.

**Methodology:** Dongying Li and Jianting Zhu.

**Software:** Runmin Li, Liping Liu, Jianting Zhu.

**Supervision:** Donying Li, Xianhuang Li.

**Writing – original draft:** Ying Li and Runmin Li.

**Writing – review & editing:** Dongying Li

## References

[1] Yu D Zheng W Cai H. Long-term diet quality and risk of type 2 diabetes among urban Chinese adults. Diabetes Care 2018;41:723–30. 29269510 10.2337/dc17-1626 PMC5860845

[2] Eltom MA Mohamed AHB Elrayah-Eliadarous H. Increasing prevalence of type 2 diabetes mellitus and impact of ethnicity in north Sudan. Diabetes Res Clin Pract 2018;136:93–9. 29203255 10.1016/j.diabres.2017.11.034

[3] Bullard KM Cowie CC Lessem SE. Prevalence of diagnosed diabetes in adults by diabetes type - United States, 2016. MMWR Morb Mortal Wkly Rep 2018;67:359–61. 29596402 10.15585/mmwr.mm6712a2 PMC5877361

[4] Kaiser AB Zhang N Der Pluijm WV. Global prevalence of type 2 diabetes over the next ten years (2018-2028). Diabetes 2018;67: Supplement 1: 202-LB.

[5] Zhang P Gregg E. Global economic burden of diabetes and its implications. Lancet Diabetes Endocrinol 2017;5:404–5. 28456417 10.1016/S2213-8587(17)30100-6

[6] Yang W. Epidemic characteristics and trend of diabetes in China. Chin Sci Life Sci 2018;48:812–9.

[7] Norhammar A. Diabetes and cardiovascular mortality: the impact of sex. Lancet Diabetes Endocrinol 2018;6:517–9. 29752193 10.1016/S2213-8587(18)30111-6

[8] Wake AD. Antidiabetic effects of physical activity: how it helps to control type 2 diabetes. Diabetes Metab Syndr Obes 2020;13:2909–23. 32884317 10.2147/DMSO.S262289 PMC7443456

[9] Institute for Health Metrics and Evaluation (IHME). Findings from the Global Burden of Disease Study 2017. Seattle, WA: IHME; 2018.

[10] Bommer C Heesemann E Sagalova V. The global economic burden of diabetes in adults aged 20-79 years: a cost-of-illness study. Lancet Diabetes Endocrinol 2017;5:423–30. 28456416 10.1016/S2213-8587(17)30097-9

[11] Chaudhury A Duvoor C Reddy Dendi VS. Clinical review of antidiabetic drugs: implications for type 2 diabetes mellitus management. Front Endocrinol (Lausanne) 2017;8:06. 10.3389/fendo.2017.00006 PMC5256065 28167928

[12] Cobbold C. Type 2 diabetes mellitus risk and exercise: is resistin involved? J Sports Med Phys Fitness 2019;59:290–7. 29498254 10.23736/S0022-4707.18.08258-0

[13] Medagama A Galgomuwa M. Comorbidities and ethnocultural factors limit the physical activity of rural Sri Lankan patients with diabetes mellitus. J Diabetes Res 2018;2018:01–6. 10.1155/2018/4319604 PMC5859883 29693020

[14] King AC Powell KE Kraus WE. The US physical activity guidelines advisory committee report-introduction. Med Sci Sports Exerc 2019;51:1203–5. 31095076 10.1249/MSS.0000000000001946

[15] Herriott MT. Effects of 8 weeks of flexibility and resistance training in older adults with type 2 diabetes. Diabetes Care 2004;27:2988–90. 15562222 10.2337/diacare.27.12.2988

[16] Wormgoor SG Dalleck LC Zinn C. Effects of high-intensity interval training on people living with type 2 diabetes: a narrative review. Can J Diabetes 2017;41:536–47. 28366674 10.1016/j.jcjd.2016.12.004

[17] Gillen JB Gibala MJ. Interval training: a time-efficient exercise strategy to improve cardiometabolic health. Appl Physiol Nutr Metab 2018;43:iii–v. 10.1139/apnm-2018-0453 30255712

[18] Warner SO Yao MV Cason RL. Exercise-induced improvements to whole body glucose metabolism in type 2 diabetes: the essential role of the liver. Front Endocrinol (Lausanne) 2020;11:567. 32982968 10.3389/fendo.2020.00567 PMC7484211

[19] Fan S Wang D Wu C. Effects of 4 major brain protection strategies during aortic arch surgery. Medicine 2018;97:11448. 10.1097/MD.0000000000011448 PMC6076180 29979447

[20] Liang J Li J Wu JY. Effectiveness comparisons of various psychosocial therapies for children and adolescents with depression: a Bayesian network meta-analysis. Eur Child Adolesc Psychiatry 2020. 10.1007/s00787-020-01492-w 32076871

[21] Thorlund K Mills EJ. Sample size and power considerations in network meta-analysis. Syst Rev 2012;1:41. 22992327 10.1186/2046-4053-1-41 PMC3514119

[22] Hutton B Salanti G Caldwell DM. The PRISMA extension statement for reporting of systematic reviews incorporating network meta-analyses of health care interventions: checklist and explanations. Ann Intern Med 2015;162:777–84. 26030634 10.7326/M14-2385

[23] Alberti KG Zimmet PZ. Definition, diagnosis and classification of diabetes mellitus and its complications. Part 1: diagnosis and classification of diabetes mellitus provisional report of a WHO consultation. Diabet Med 1998;15:539–53. 9686693 10.1002/(SICI)1096-9136(199807)15:7<539::AID-DIA668>3.0.CO;2-S

[24] American Diabetes Association. 1. Improving care and promoting health in populations: standards of medical care in diabetes-2020. Diabetes Care 2020;43: Suppl 1: S7–13. 31862744 10.2337/dc20-S001 PMC11869376

[25] Chinese guidelines for the prevention and treatment of type 2 diabetes mellitus (2017, Edition). Chin J Diabetes 2018;10:04–67.

[26] Cumpston M Li T Page MJ. Updated guidance for trusted systematic reviews: a new edition of the Cochrane Handbook for Systematic Reviews of Interventions. Cochrane Database Syst Rev 2019;10:ED000142. 31643080 10.1002/14651858.ED000142 PMC10284251

[27] Stroup DF Berlin JA Morton SC. Meta-analysis of observational studies in epidemiology: a proposal for reporting. Meta-analysis of observational studies in epidemiology (MOOSE) group. JAMA 2000;283:2008–12. 10789670 10.1001/jama.283.15.2008

[28] Stevens M King DL Dorstyn D. Cognitive-behavioral therapy for Internet gaming disorder: a systematic review and meta-analysis. Clin Psychol Psychother 2019;26:191–203. 30341981 10.1002/cpp.2341

[29] Tarsilla M. Cochrane handbook for systematic reviews of interventions. Naunyn Schmiedebergs Arch Exp Pathol Pharma-kol 2011;5:S38.

[30] Jansen JP Naci H. Is network meta-analysis as valid as standard pairwise meta-analysis? It all depends on the distribution of effect modifiers. BMC Med 2013;11:159. 23826681 10.1186/1741-7015-11-159 PMC3707819

[31] Mavridis D Salanti G. A practical introduction to multivariate meta-analysis. Stat Methods Med Res 2013;22:133–58. 22275379 10.1177/0962280211432219

[32] Page MJ Shamseer L Altman DG. Epidemiology and reporting characteristics of systematic reviews of biomedical research: a cross-sectional study. PLoS Med 2016;13:e1002028. 27218655 10.1371/journal.pmed.1002028 PMC4878797

[33] van Valkenhoef G Dias S Ades AE. Automated generation of node-splitting models for assessment of inconsistency in network meta-analysis. Res Synth Methods 2016;7:80–93. 26461181 10.1002/jrsm.1167 PMC5057346

[34] Stang A. Critical evaluation of the Newcastle-Ottawa scale for the assessment of the quality of nonrandomized studies in meta-analyses. Eur J Epidemiol 2010;25:603–5. 20652370 10.1007/s10654-010-9491-z

[35] Qin L Zhu H. Effect of 16 week walking combined with table tennis on quality of life in elderly patients with type 2 diabetes mellitus. J Guangzhou Inst Phys Educ 2016;36:109–12.

[36] Li Q. Effects of Baduanjin on lower limb muscle strength and body composition of patients with type 2 diabetes mellitus. Tianjin Tradit Chin Med 2016;33:661–4.

[37] Wu Y Wei Q. Clinical observation of Baduanjin in adjuvant treatment of type 2 diabetes mellitus. Chines J Gerontol 2015;33:5237–40.

[38] Guan Y Wang S Ma M. Baduanjin Exercise Intervention on related indicators of patients with type 2 diabetes mellitus. J Nurs 2012;27:23–4.

[39] Li ZB Qi LL Zhao L. Advantages of Baduanjin in aerobic exercise therapy for patients with type 2 diabetes mellitus. Liaoning J Tradit Chin Med 2013;40:1858–60.

[40] Lian Y. Effect of walking exercise on blood biochemical indexes of elderly patients with newly diagnosed type 2 diabetes mellitus. Chin J Geriatr Health Med 2019;17:97–8.

[41] Gao T. Effect of modified Baduanjin on quality of life in patients with type 2 diabetes mellitus. Beijing J Tradit Chin Med 2019;38:584–8.

[42] Lan P. Observation on the curative effect of jogging in the treatment of middle-aged and elderly type 2 diabetes mellitus. Chin J Gerontol 2008;28:816–7.

[43] Wang P. Evaluation of intervention effect of different aerobic exercise modes on patients with type 2 diabetes mellitus in community. China Med Guide 2009;6:34–5.

[44] Yang H. Effects of Baduanjin on clinical efficacy, psychological status and blood glucose indexes of elderly patients with type 2 diabetes mellitus. Chin J Gerontol 2019;39:3433–5.

[45] Li X. Effect of Taijiquan exercise on oxidative stress and inflammation in elderly patients with type 2 diabetes mellitus. Chin J Gerontol 2013;33:5465–6.

[46] Wei D. Research on the influence of Taiji Softball Exercise on physical fitness of patients with type 2 diabetes mellitus. J Nanjing Inst Phys Educ Nat Sci Ed 2012;11:08–11.

[47] Xue W. Research on the influence of tennis on hemorheology of type 2 diabetes mellitus. J Liaoning Normal Univ (Nat Sci Ed) 2012;35:136–9.

[48] 2005;Zhang C Xie S Gao D. Effects of uniform walking on blood pressure, blood glucose and lipid metabolism in patients with type 2 diabetes mellitus. 9:74–8.

[49] Wang D Chen X Xu G. Effects of Taijiquan combined with Baduanjin on blood glucose and quality of life in community patients with type 2 diabetes mellitus. J Nurs 2016;31:37–9.

[50] Li B. Effects of moderate intensity exercise on insulin sensitivity and plasma adiponectin level in patients with type 2 diabetes mellitus and obesity. Chin J Clin Rehabil 2005;39:22–4.

[51] Feng W Mao J Bao W. Comparison of therapeutic effects of different exercise therapies on type II diabetes. J Kunming Med Univ 2018;39:43–6.

[52] Colberg SR Hagberg JM Mccole SD. Utilization of glycogen but not plasma glucose is reduced in individuals with NIDDM during mild- intensity exercise. J Appl Physiol 1996;81:2027–33. 8941525 10.1152/jappl.1996.81.5.2027

[53] Fujian University of Traditional Chinese Medicine, Ye X. A Network Meta-analysis of the Effects of Seven Aerobic Exercise Therapies on Type 2 Diabetes Mellitus. 2019.

[54] Dintenfass L. Rheology of Blood in Diagostic and Preventive Medicine. 1976;London: Butterworths, 31.

